# Inhibition of RNA Helicases of ssRNA^+^ Virus Belonging to Flaviviridae, Coronaviridae and Picornaviridae Families

**DOI:** 10.1155/2011/213135

**Published:** 2010-11-14

**Authors:** Irene Briguglio, Sandra Piras, Paola Corona, Antonio Carta

**Affiliations:** Department of Medicinal and Toxicological Chemistry, University of Sassari, Via Muroni 23/a, 07100 Sassari, Italy

## Abstract

Many viral pathogens encode the motor proteins named RNA helicases which display various functions in genome replication. General strategies to design specific and selective drugs targeting helicase for the treatment of viral infections could act *via* one or more of the following mechanisms: inhibition of the NTPase activity, by interferences with ATP binding and therefore by limiting the energy required for the unwinding and translocation, or by allosteric mechanism and therefore by stabilizing the conformation of the enzyme in low helicase activity state; inhibition of nucleic acids binding to the helicase; inhibition of coupling of ATP hydrolysis to unwinding; inhibition of unwinding by sterically blocking helicase translocation. Recently, by *in vitro* screening studies, it has been reported that several benzotriazole, imidazole, imidazodiazepine, phenothiazine, quinoline, anthracycline, triphenylmethane, tropolone, pyrrole, acridone, small peptide, and Bananin derivatives are endowed with helicase inhibition of pathogen viruses belonging to Flaviviridae, Coronaviridae, and Picornaviridae families.

## 1. Introduction

To convert a closed double-stranded DNA or RNA helix into two open single strands, so that other protein machinery can manipulate the polynucleotides, the cells require helicases. They are motor proteins that use energy derived from ATP hydrolysis [1–4]. Several DNA and RNA helicases have been isolated from all kingdoms of life, from virus to man [5–8]. Detailed structural information, biological mechanisms, and clear outlook on inhibitors of therapeutic relevance as antiviral agents are recently provided by Xi et al. [9], Kwong et al. [10], and overall Frick et al. [11, 12]. 

Several ssRNA^+^ (positive sense single-stranded RNA) helicases have been studied in detail including those from *Dengue fever virus* (DFV), *West Nile virus* (WNV), and *Japanese encephalitis virus* (JEV). More in general, a recent article on anti-Flaviviridae chemotherapy has been published by Ghosh and Basu [13], who expand the original information regarding the role of helicases in *Flaviviridae* previously reported by Borowski [14]. 

This enzyme is a promising target to develop new therapies and preventative agents, since ssRNA^+^ viruses belonging to families like Flaviviridae, Coronaviridae, and Picornaviridae cause clinically significant diseases both in humans and animals, determining life lost, economical loss, and higher productivity costs. Examples are the bovine viral diarrhea virus (BVDV), a serious welfare problem that significantly damages the farm business, and the Hepatitis C virus [HCV], that is now a global public health issue, being a major cause of human hepatitis [15]. Actually, with the exception of YFV, no vaccine exists against the various *Flaviviridae* members therefore, new therapies and preventative agents are strongly needed.

Viruses belonging to *Picornaviridae* family cause a variety of illnesses, including meningitis, cold, heart infection, conjunctivitis, and hepatitis [16]. This family includes nine genera, some of which comprise major human pathogens, namely, *Enterovirus* (including *Poliovirus*, *Coxsackievirus*, *Echovirus*), *Rhinovirus* (approximately 105 serotypes), and *Hepatovirus* (Hepatitis A virus). At present, no specific antiviral therapy is available for the treatment of Picornaviridae infections.

Finally, Severe Acute Respiratory Syndrome *Coronavirus* (SARS-CoV), an enveloped virus, has recently infected thousand of humans, with about 800 deaths, and no vaccine or specific antiviral therapy is known against this virus.

No retroviruses or ssRNA^−^ viruses have been reported to encode the synthesis of a helicase; they might simply utilize helicases encoded by the host cell instead of their own proteins, as recently shown for HIV replication, which requires the human DDX3 DEAD-box RNA helicase [17, 18].

In ssRNA^+^ viruses, the RNA helicases are implicated in several functions including RNA genome replication, ribosome biogenesis, messengers RNA transcription, pre-mRNA splicing, RNA maturation, RNA export and degradation, as well as RNA translation [19, 20]. 

Basing on certain signature motifs in the amino acid sequence, Gorbalenya and Koonin have shown that all helicases can be classified in several genetic families [21]. All but two of the helicase families can be grouped into one of three larger “superfamilies,” designed as superfamily 1 (**SF1**), superfamily 2 (**SF2**) [22], and superfamily 3 (**SF3**) [23].

Of the remaining 2 families, one is similar to the DnaB helicase of *E. coli* [5] and the other resembles the *E. coli* Rho helicase that is used in transcriptional termination [24]. Only the DnaB-like family, sometimes called family 4 (F4) or superfamily 4 (**SF4**), contains viral proteins [25]. 

**Figure 1:**
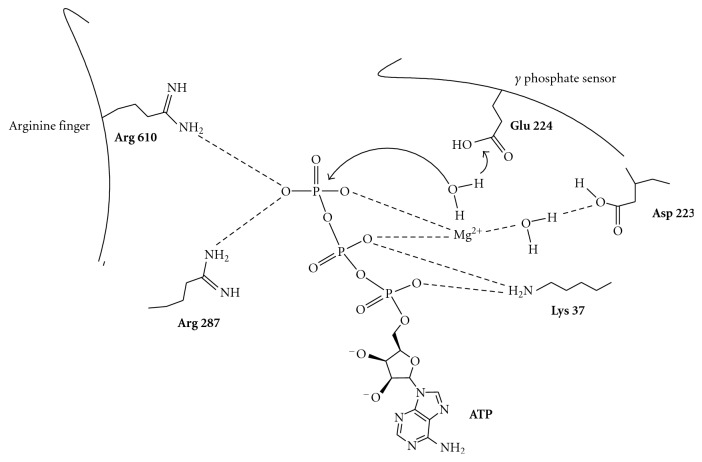
Mechanism of helicase-catalyzed ATP hydrolysis. Helicases coordinate an ATP, Mg^2+^ and a water molecule using a conserved Lys and Asp in the Walker A and B motifs on one RecA-like domain and an Arg on an adjacent RecA-like domain. A Glu likely acts as a catalytic base by accepting a proton from the attacking water molecule [11].

All helicases bind NTP using two structurally common amino acidic sequences named motif I and motif II, described by Walker et al. [25] and Subramanya et al. [26]. Motif I (also known as Walker A motif/boxes A) is a phosphate-binding P-loop that also interact with the ribose, while motif II (also known as Walker B/boxes B) is a Mg^2+^ co-factor binding loop. The ATP-binding site of helicase is completed by an arginine “finger” and a catalytic base, which accepts a proton from the attacking water molecule. In related proteins, this catalytic base has been demonstrated to be a conserved glutamate near the Walker B motif [27, 28]. Arginine amino acids often interact with the beta and gamma phosphates of the bound ATP, stabilizing the transition state [29, 30], Figure 1.

All helicases can also be classified according to their movement relative to the nucleic acid strand to which they are primarily associated or to their quaternary structures.

Thus, a helicase can be classified basing on each of the three above schemes. For example, the helicase encoded by HCV (Hepatitis C Virus) is an **SF2**, nonring, 3′–5′ RNA helicase. Human papillomavirus helicase is an **SF3**, ring, 3′–5′ DNA helicase. 

The functional importance of helicases means that inhibitors or modulators for these enzymes are potentially important as therapeutic agents. Over the past decade, significant progress has been made in the development of highly selective inhibitors as antiviral and anticancer drugs for clinical uses. Developing nontoxic helicase inhibitors as antiviral drugs is considerably more difficult than developing drugs designed to inhibit other viral enzymes. In fact, in contrast with proteases and polymerases, the helicases-dependent reactions are still not fully elucidated. Furthermore, the helicase ATP-binding site is conserved not only in all the classes of helicases, but also in other proteins necessary for the cellular lifecycle, such as small GTPases, kinases, the AAA^+^ family (ATPases associated with various cellular activities), and even the mitochondrial ATP synthase (F1 ATPase). Thus, compounds that inhibit helicases *via* their ATP-binding sites could have toxic effects on the host cells.

## 2. Viral RNA Helicases As Antiviral Drug Targets

Many viral pathogens encode RNA helicases which have been demonstrated essential for viral replication and pathogenesis [31–33]. Between them are

- emerging or re-emerging viruses with pandemic potential, such as *SARS-Cov* (Severe Acute Respiratory Syndrome-Coronavirus), *Dengue*, *West Nil*e, and *Japanese encephalitis viruses*,
- viruses that have a stable spread worldwide, such as HCV (*Hepatitis C Virus*),
- viruses that do not have a large spread, but can generate serious health problems because of lack or limited availability of effective drugs, such as CVB (*Human Coxsackie B Virus*).

General strategies to design specific and selective drugs for the treatment of viral infections targeting helicase could act *via *one or more of the following mechanisms:

1. inhibition of the NTPase activity by interferences with ATP binding and therefore by limiting the energy required for the unwinding and translocation,
2. inhibition of the NTPase activity by allosteric mechanism and therefore by stabilizing the conformation of the enzyme in low helicase activity state,
3. inhibition of nucleic acids binding to the helicase,
4. inhibition of coupling of ATP hydrolysis to unwinding,
5. inhibition of unwinding by sterically blocking helicase translocation,
6. development of small molecule antagonists against essential protein-protein interactions involving helicases.

Some characteristics of helicase families of pathogen viruses belonging to Flaviviridae, Coronaviridae, and Picornaviridae families are reported in Table 1 [9, 10, 34].

## 3. Flaviviridae

The Flaviviridae is a large family of related positive-strand RNA viruses that currently consists of three genera: Flavivirus, Pestivirus (from the Latin *pestis*, plague), and Hepacivirus (from the Greek *hepatos*, liver). In addition, the family includes two groups of viruses, GBV-A and GBV-C, that are currently unassigned to a specific genus and await formal classification [35]. Within this family are comprised viruses that cause significant diseases in human and animal populations. From *Flavivirus* genus is Dengue virus (DENV) with its associated dengue hemorrhagic fever (DHF) and dengue shock syndrome (DSS), Japanese encephalitis virus (JEV), West Nile virus (WNV), Yellow Fever virus (YFV), and tick-borne encephalitis virus (TBEV). The *Pestivirus*es are animal pathogens of major economic importance for the livestock industry, like bovine viral diarrhea virus (BVDV), border disease virus (BDV) of sheep, and classical swine fever virus (CSFV). The *Hepacivirus* genus includes only the hepatitis C virus (HCV), an important human pathogen. 

HCV, identified in 1989 [36], is a major cause of human hepatitis, globally, and infects about 3% of the world's population [37]. Hepacivirus is spread primarily by direct contact with human blood; hence, the major causes of infection are use of unscreened blood transfusions and reuse of needles and syringes that have not been adequately sterilised. The World Health Organization (WHO) estimates that over 170 million people worldwide are presently infected with this virus [38]. Most infections become persistent and about 60% of cases progress towards chronic liver disease, that can lead to development of cirrhosis, hepatocellular carcinoma, and liver failure [39, 40]. 

Pegylated interferon in combination with ribavirin is used in the clinic for hepatitis due to HCV. Unfortunately, this therapy requires lengthy periods of administration and is often associated with severe and adverse events. Furthermore, this drug has limited efficacy and the sustained virological response rate is of 40–50% in genotype HCV-1 infected patients, and of 80% in those infected with genotypes 2 and 3 [41, 42]. 

This emphasizes that new therapies are clearly needed, since for the treatment of this infection, and generally for diseases caused by viruses belonging to the Flaviviridae family, therapeutic strategies really effective and selective are not available. 

**Figure 2:**
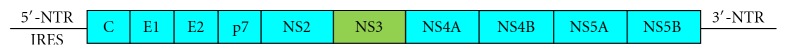
Simplified representation of structure of Hepacivirus and Flaviviruses polyprotein.

All of the 12 HCV genotypes, which have nucleotide sequences that differ by as much as 30%, produce a single polyprotein of about 3,000 amino acids, which is subsequently processed by viral and host proteases into four structural proteins and six nonstructural proteins (altogether 10 mature proteins). As summarized in Figure 2, the structural proteins (*S proteins*: core, E1, E2, and p7) generate the viral capsid and envelope proteins and are cleaved by host-signal peptidases, while the six nonstructural proteins (*NS proteins*: NS2, NS3, NS4A, NS4B, NS5A, and NS5B) are responsible for genome replication and are largely generated by HCV-encoded protease [43]. 

HCV Helicase is part of the bi-functional NS3 protein, carrying three different enzymatic activities: helicase, NTPase, and serine protease activities. 

NS3 helicase is essential for viral replication, and this makes it one of the most promising target for the antiviral therapy.

The known HCV helicase inhibitors can be classified on the base of their mechanism of action, into the first four groups of those above cited:

1. inhibitors of NTPase activity by interference with NTP binding,
2. inhibitors of NTPase activity by allosteric mechanism,
3. competitive inhibitors of RNA binding,
4. inhibitors of the coupling of NTP hydrolysis at the unwinding reaction.

### 3.1. Inhibition of NTPase Activity by Interference with NTP Binding

The hydrolysis of ATP supplies the energy that allows the helicase to adopt various nucleotide ligation states that allosterically cause conformational changes in the nucleic acid binding site to drive the movement of the helicase along the length of the nucleic acid chain [19]. So, competitive NTPase inhibitors may lead to decreased ATPase activity and therefore to reduction of the unwinding rate. 

**Figure 3:**
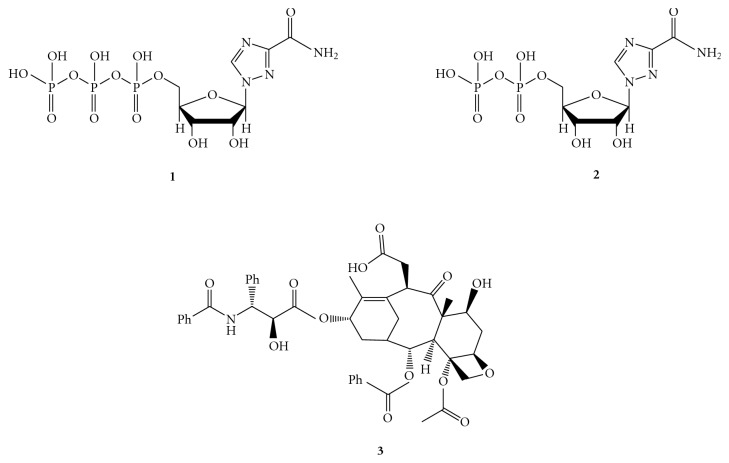
Structure of three competitive HCV helicase inhibitors ribavirin 5′-triphosphate (**1**), ribavirin 5′-diphosphate (**2**), and paclitaxel (**3**).

Consequently, non-(or slowly) hydrolysable ATP-analogs seemed to be effective tools for inhibiting the helicase activity, like adenosine-5′*γ*-thiotriphosphate (ATP-*γ*-S), which is used to determine a low level of unwinding of HCV dsRNA [44, 45]. However, ribavirin 5′-triphosphate (RTP), that inhibits the HCV NTPase/helicase by a competitive mechanism in regard to ATP [46], and ribavirin 5′-diphosphate (RDP), both reported in Figure 3, even showing IC_50_ values in the micromolar range, demonstrates to determine only a weakly enzymatic inhibition [34]. The same behavior has been put in evidence for paclitaxel, compound structurally nonrelated to NTP. This derivative is able to block the NTP-binding site (IC_50_ = 22 *μ*M) and to inhibit the ATPase activity (IC_50_ = 17 *μ*M) in a competitive way, but is not able to inhibit the helicase activity at concentration lower than 1 mM [14] 

The partial unwinding activity mediated by these competitive NTPase inhibitors is common to all members of the class, and the concentrations needed for the helicase inhibition usually exceed the IC_50_ value by 3–5 times. At these concentrations, the NTPase activity reached 10–35% of the control [46–48]. The basis for the phenomenon remains unclear.

**Figure 4:**
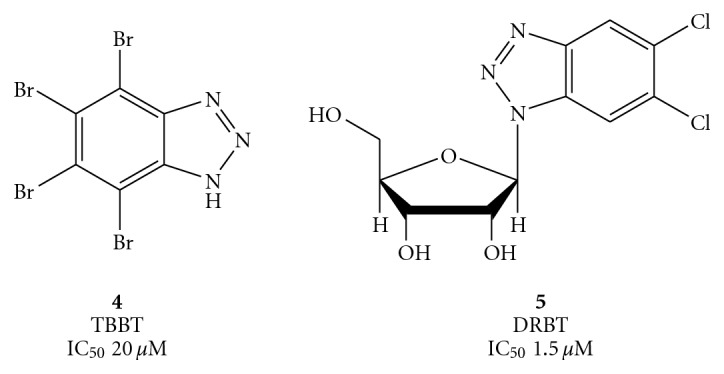
Structure of the halogenated benzotriazoles TBBT (**4**) and DRBT (**5**).

On the other hand, most potent benzotriazole helicase inhibitors were identified during the course of a random screening study [49, 50]. In particular, 4, 5, 6, 7-tetrabromobenzotriazole (TBBT) (**4**), known as a potent and highly selective inhibitor of protein kinase 2, and 5,6-dichloro-1-(*β*-D-ribofuranosyl) benzotriazole (DRBT) (**5**) displayed IC_50_ values of 20 and 1.5 *μ*M, respectively (Figure 4).

On the contrary, the corresponding imidazole derivative of DRBT, the 5, 6-dichloro-1-(*β*-D-ribofuranosyl) benzimidazole (DRBI), against NTPase/helicase of a large number of members of the Flaviviridae family (HCV, WNV, DENV, and JEV) resulted to be completely inactive.

**Figure 5:**
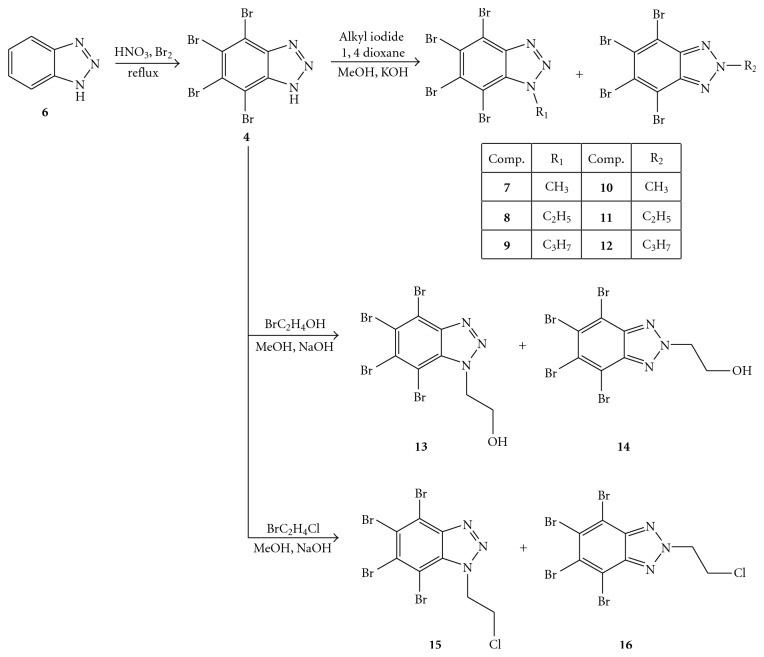
Synthesis of TBBT (**4**) and its *N*-alkyl derivatives.

**Figure 6:**
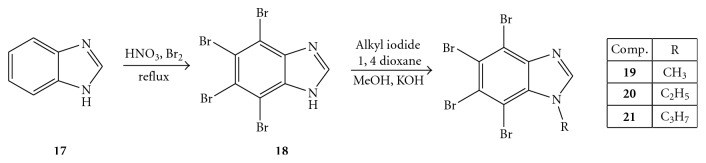
Synthesis of 4, 5, 6, 7-tetrabromo 1*H*-benzimidazole.

To explain this finding, Bretner et al. synthesized and studied a new series of substituted (alkyl, hydroxy alkyl, chloro alkyl, ribofuranose) 1*H*-benzimidazole and 1*H*-benzotriazole derivatives shown in Figures 5 and 6 [50, 51]. 

TBBT (more less DRBT) resulted effective in HCV subgenomic replicon system in a comparable way to the inhibition reported in the enzymatic essays, showing a property that has been detected only for a handful group of HCV inhibitors [52]. 

It has been reported that the starting compounds 1*H-*benzotriazole (**6**) and 1*H-*benzimidazole (**17**), screened for their effect against the HCV-helicase, showed

- very low activity (IC_50_ 200 *μ*M and 500 *μ*M, respectively) when measured with a DNA substrate,
- no activity when measured either with an RNA substrate or against the flavivirus enzymes of WNV, DENV, and JEV (IC_50_> 500 *μ*M).

On the contrary, the whole halogenation of 1*H-*benzotriazole (**6**) with bromine atoms, to afford the above cited **4**, caused either a 10-fold or 9-fold more effective inhibition of the HCV helicase when determined with a DNA substrate or an RNA substrate, respectively, and of 25-fold in the case of the JEV enzyme (IC_50_ 20 *μ*M). 

The corresponding bromination of 1*H-*benzimidazole (**17**) afforded the derivative (**18**), which resulted to be less effective than **4** and 2–2.5 times more potent than parent **17** against HCV helicase.

When 1- or 2-alkyl benzotriazoles were screened for their effect on the HCV-helicase activity using the DNA substrate, the 2-alkylated derivatives (**10**–**12**) resulted to be significantly more potent inhibitors of the enzyme (2- to 7- times) than the respective 1-alkylated analogues (**7**–**9**). 

On the other hand, enhancement of the activity was observed when the aliphatic chain was elongated in both 1-alkylated benzotriazoles (**7**–**9**) and 1-alkylated benzimidazoles (**19**–**21**) than the respective 2-alkylated analogues. In the case of the benzimidazole derivatives (**19**–**21**), however, the inhibitory activity was very low and ranged between 250 and 500 *μ*M. Furthermore, the HCV helicase activity of the alkylated benzimidazoles tested using the RNA substrate, as well as using other viral NTPase/helicases, displayed no inhibitory activity. 

This behaviour suggests that these inhibitors do not act by blocking the NTP binding sites of the enzymes and that occupation of an allosteric nucleoside binding site should be considered, as previously suggested by Porter [53]. 

Furthermore, in this study the authors observed that replacement of the alkyl side-chain by a substituent endowed with higher hydrophilicity (hydroxyethyl derivatives **13** and** 14** in Figure 5) or with higher hydrophobicity (chloroethyl derivatives** 15** and **16** in Figure 5) dramatically decreases the activity of the tetrabromobenzotriazoles. Consequently, it seems that a small hydrophobic alkyl moiety (methyl or ethyl) at position 2- of the tetrabromobenzotriazole could play a crucial role in the inhibition of the HCV NTPase/helicase. 

**Figure 7:**
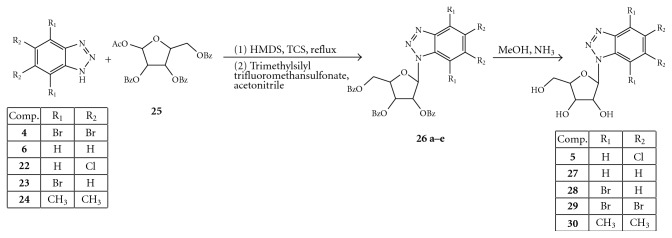
Synthesis of compounds **5, 27**–**30**.

Introduction of a ribofuranosyl ring in both benzotriazole and tetrabromobenzotriazole improves the water solubility but leads to a decrease of the inhibitory activity against HCV and all the enzymes tested. The same substituent in the position 1 of the 5,6-dichlorobenzotriazole DRBT (**5**) was, as above reported, very effective in inhibiting the HCV and WNV helicases (IC_50_ 1.5 *μ*M and 3.0 *μ*M, respectively) but ineffective against JEV helicase [49]. On the contrary, replacement of chlorine atoms of the benzotriazole ring with either bromine atoms or methyl groups (compounds **28**–**30**, Figure 7) showed lower activity compared to DRBT. 

**Figure 8:**
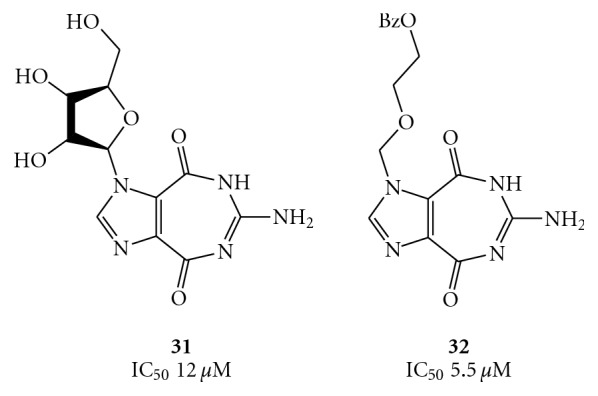
Structures of the ring expanded nucleosides **31** and **32**.

In an extension of this study, an additional class of nucleoside analogues known as ring-expanded nucleosides (REN or “fat”) involving 6-aminoimidazo [4,5-*e*] [1, 3] diazepine-4,8-dione ring were reported to be active against the helicase unwinding reaction [54]. A number of RENs, such as compounds **31 **and **32 **of Figure 8, displayed IC_50_ values in the micromolar range. In view of the observed tight complex between some nucleosides and RNA and/or DNA substrates of a helicase, the mechanism of REN action might involve binding to the minor or major groove of the helical nucleic acid substrate. 

The fat nucleosides **31**, **32,** and TBBT (**4**) and nogalamycin (see compound **76**) have been recently used to construct a pharmacophore model for designing new Japanese encephalitis virus NS3 helicase/NTPase inhibitors, using a refined structure of this enzyme [55]. 

**Figure 9:**
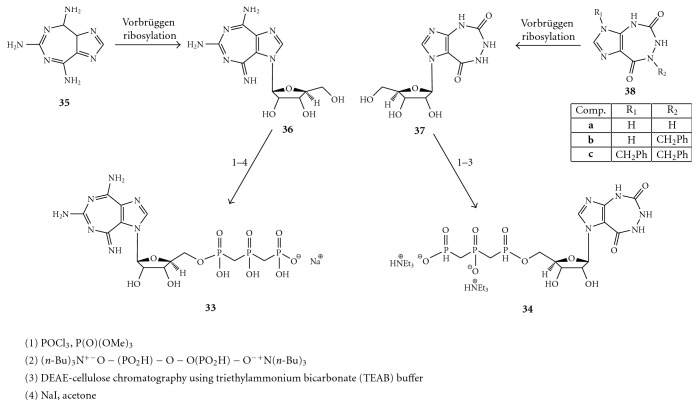
Synthesis of compounds **33** and **34**.

On the other hand, the REN 5′-triphosphates, such as compounds **33 **and **34** of Figure 9, did not influence the unwinding reaction while exerting their inhibitory effect (IC_50_ 0.55 *μ*M and 1.5 *μ*M, respectively) on the ATPase activity of the enzyme. As reported in Figure 9, compounds **33** and **34**, containing the 5 : 7-fused heterocyclic systems, imidazo [4,5-*e*] [1, 3] diazepine and imidazo [4,5-*e*] [1, 2, 4] triazepine, respectively, were synthesized from the corresponding nucleosides **36** and **37**, employing the Ludwig's procedure [56]. The nucleosides **36 **and **37**, in turn, were synthesized by Vorbrüggen ribosylation [57–60] of the respective heterocycles **35 **and **38 **[61, 62].

Therefore, in exploring the potential anti-Flaviviridae activity of the ring system contained in **31, **the same authors focused on different substituents (alkyl, arylalkyl, and aromatic groups) at position 6, along with variations of sugar moieties at position 1 (ribose, 2′-deoxyribose, or acyclic derivatives) as well as their attachment to the base (*α* or *β* configuration) [63].

**Figure 10:**
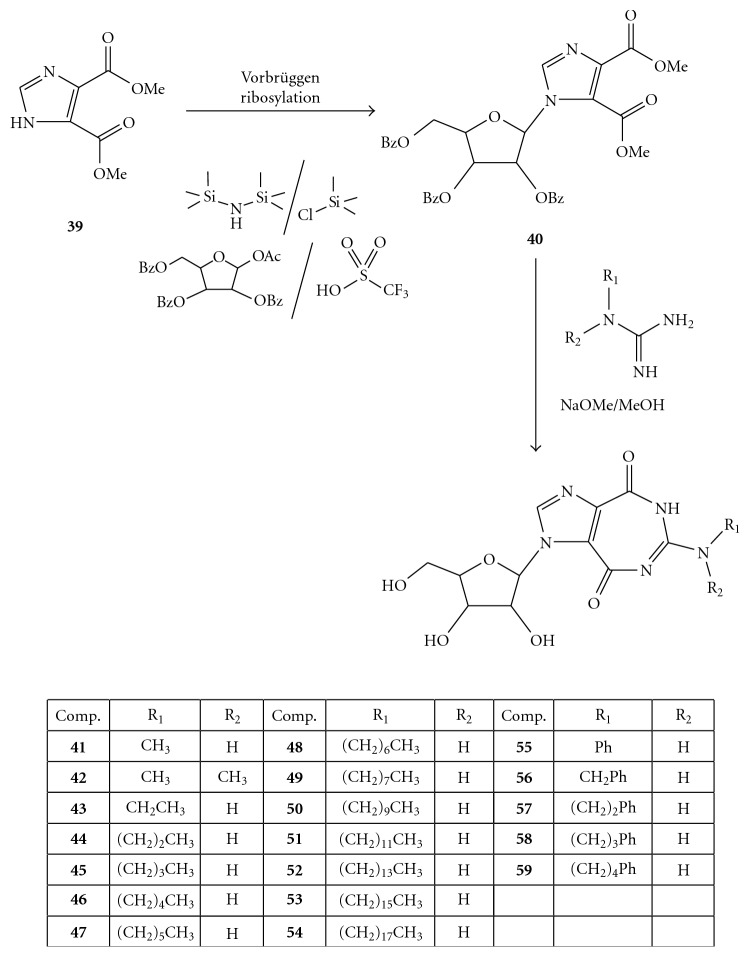
Synthesis of the compounds **41**–**59**.

The general method for the synthesis of the designed nucleosides (**41**–**59**) was involved, as reported in Figure 10, the Vorbrüggen ribosylation [53, 54] of dimethyl imidazole-4,5-dicarboxylate (**39**) [64, 65], followed by condensation of the resulting imidazole nucleoside (**40**) with the appropriately substituted guanidine derivatives.

The modulation effect exerted by RENs can result in an inhibition or activation. In the first case, the mechanism may involve the interaction of RENs with a DNA or an RNA substrate through binding to the major or minor groove of the double-helix. In the case of activation, the mechanism may involve an allosteric binding site that can be occupied by nucleoside/nucleotide-type molecules including, but not limited to RENs. The occupation of this allosteric site on the enzyme is dependent upon the high level of ATP (NTP) concentration in the reaction mixture. 

**Figure 11:**
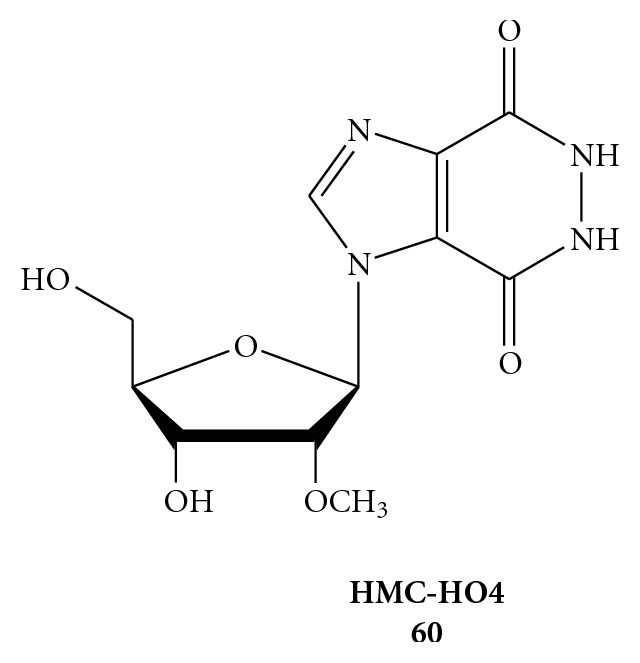
Chemical structure of 1-(2′-O-Methyl-*β*-D-ribofuranosyl)imidazo[4,5-d]pyridazine-4, 7(5*H*, 6*H*)-dione (HMC-HO4) (**60**).

RENs obtained with the above procedures were screened for inhibition of NTPase/helicase of the WNV. One of the most promising among these early inhibitors is 1-(2′-O-methyl-*β*-D-ribofuranosyl)imidazo[4,5-*d*]pyridazine-4,7(5*H*,6*H*)-dione (HMC-HO4) (**60**), Figure 11, which produces a promising antiviral effect (EC_50_ = 25–30 *μ*M) [66]. At all the concentrations of HMC-HO4, ATP hydrolysis is stimulated, suggesting that the inhibitor somehow uncouples the ATPase and helicase functions. In that regard, RENs may represent a starting point for the development of highly selective inhibitors of WNV NTPase/helicase.

**Figure 12:**
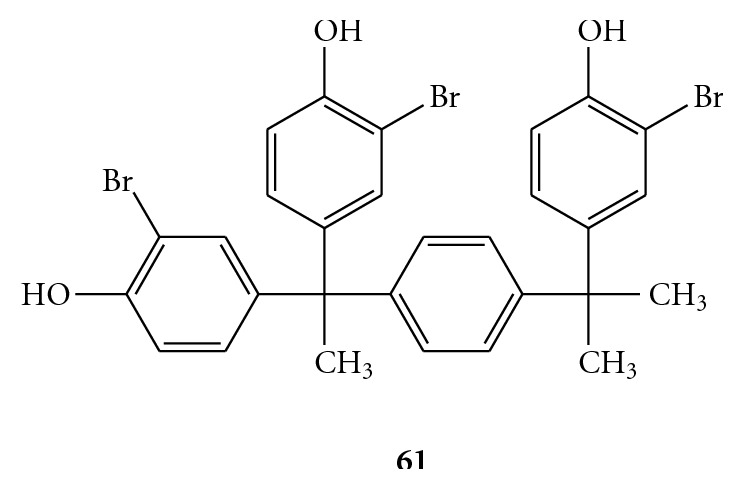
Chemical  structure  of  4, 4′-(1-(4-(2-(3-bromo-4-hydroxyphenyl)propan-2-yl)phenyl)ethane-1,1-*di*yl)bis(2-bromophenol) (**61**).

An other recent starting point is represented by triphenylmethane derivatives, as reported from Chen et al. [67]. Compound (**61**) of Figure 12, where the triphenylmethane moiety is linked to a 2-(3-bromo-4-hydroxyphenyl)propane, was identified as a good inhibitor that suppresses HCV RNA replication in the HCV replicon cells through both the inhibition of ATP hydrolysis and the RNA substrate binding [67].

### 3.2. Inhibition of NTPase Activity by Allosteric Mechanisms

**Figure 13:**
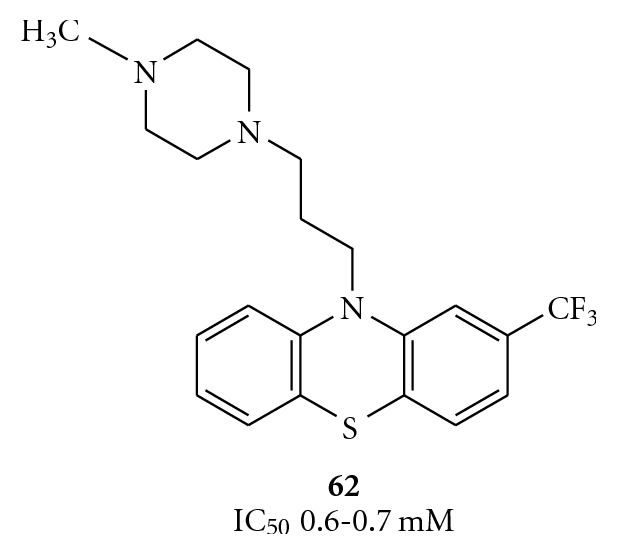
Structure of the calmodulin antagonist trifluoperazine (**62**).

The partial inhibition mediated by the competitive NTPase inhibitors may be avoided by utilizing compounds chemically unrelated to NTP, which reduce the accessibility to the NTP-binding site in a noncompetitive manner [68]. An example is the calmodulin antagonist trifluoperazine (**62**, Figure 13). Although the molecule is known to interact with domain 1 of HCV helicase, it is uncertain if inhibition results from conformational changes or from blockage of the ATP-binding site [46]. 

Even some tropolones have been screened as inhibitors of HCV helicase-catalyzed DNA unwinding. Recently Bernatowicz et al. have described several derivatives bearing a side chain that connect the seven-member ring system to some N-heterocycles.

**Figure 14:**
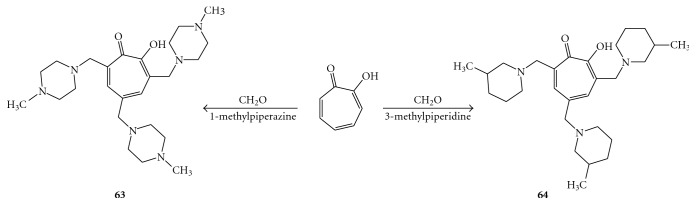
Structure and synthesis of the compounds **63** and **64**.

The  most  active  compound,  3,5,7-tri[(40-methylpiperazin-10-yl)methyl]tropolone  (**63**),  inhibited RNA replication by 50% at 46.9 *μ*M (EC_50_), showing an IC_50_ = 3.4 *μ*M and a CC_50_> 1000 *μ*M (SI > 21), whereas the most efficient was 3,5,7-tri[(30-methylpiperidin-10-yl)methyl]tropolone (**64**), with an EC_50_ of 35.6 *μ*M, which unfortunately exhibited a lower SI (9.8) derived by a CC_50_ = 348 *μ*M. These tropolone derivatives, reported in Figure 14, are the first antihelicase compounds that inhibit HCV replication with the ability to cause the appearance of resistant mutants, suggesting that inhibition of replication is the result of inhibition of the enzyme activity. They also inhibit replication of the HCV subgenomic replicon in cell cultures [69].

### 3.3. Competitive Inhibition of RNA Binding

Several polynucleotides displayed inhibitory HCV helicase activity. The inhibition is believed to result from the competition of the polynucleotides with DNA or RNA substrates, an effect that could be mimicked by synthetic macromolecules [46].

**Figure 15:**
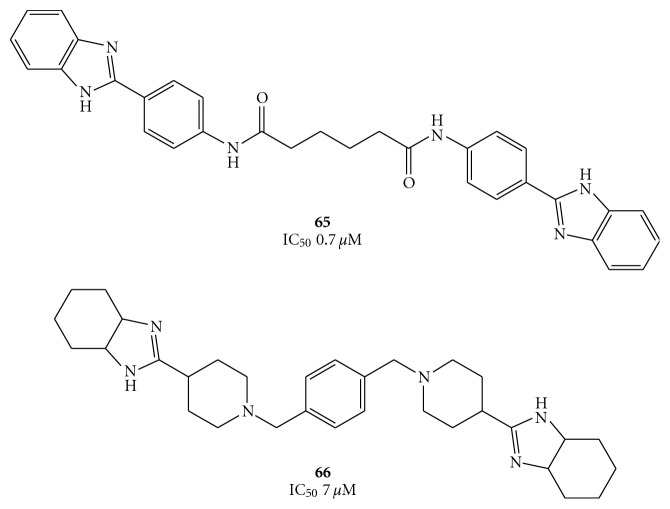
The HCV helicase inhibitors reported by ViroPharma Inc.

With the aim of discovering new anti-HCV agents, ViroPharma synthesized several benzimidazole derivatives, two of them (compounds **65** and **66**, Figure 15) showing high activity against HCV helicase [70]. Although the exact mechanism of **65** and **66** is still not clear, they might compete with nucleic acids in the manner above mentioned. In particular, the benzene ring and the NH group could interact by hydrophobic interaction and hydrogen bound, respectively. 

**Figure 16:**
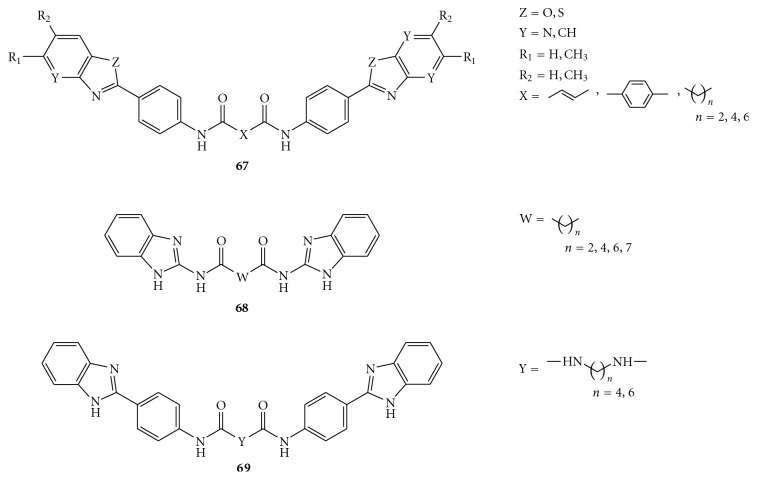
Structures of diamides (**67**), aminobenzimidazole-derived diamides (**68**), and two aminophenyl benzimidazole-derived diureas (**69**).

In the attempt to extend the SAR analysis, some new dimers containing benzimidazole, benzoxazole, pyridinoxazole, and benzothiazole rings, attached to symmetrical linkers, were synthesized by Phoon et al., as summarized in Figure 16 [70, 71]. Preliminary studies of these compounds showed a significant decrease in potency when the benzimidazole moiety was replaced by the benzoxazole or benzothiazole rings (compounds **67**). On the other hand, the aminobenzimidazole-diamides (**68**) and aminophenyl benzimidazole-diureas (**69**) derivatives displayed, at 25 *μ*g/mL, 6–13, and 20–28 percent inhibitory activity, respectively. 

Likewise, the linker was also implicated in the inhibitory activity since replacement of the diamide linkage possessed by **65 **with the diurea linkage (compounds **69**) led to reduced potency. Thus, the SAR data indicate that the benzimidazole ring, the benzene group at the C2 position of the benzimidazole moiety, and the nature of the linker are essential for the activity [70].

**Figure 17:**
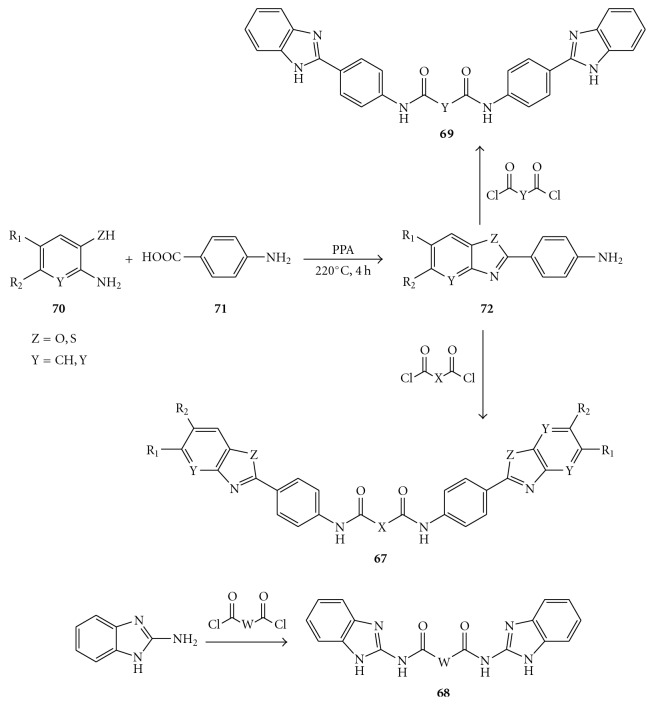
Synthesis of the diamides (**67**), aminobenzimidazole-derived diureas (**68**), and aminophenyl benzimidazole-derived diamides (**69**).

The synthesis of these analogues is outlined in Figure 17. Aminophenols and thiophenols, or the corresponding pyridine derivatives, reacted easily with *p*-aminobenzoic acid in the presence of polyphosphoric acid to afford the corresponding oxazole and thiazole derivatives (**72**). Subsequent coupling of **72 **and 2-aminobenzoimidazole with the opportune acid dichlorides furnished the products **67**–**69**.

**Figure 18:**
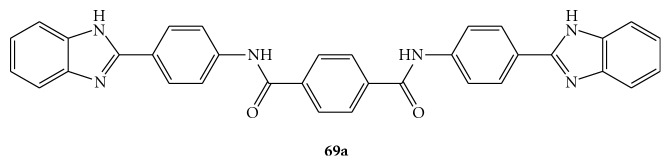
Symmetrical benzimidazolephenylcarboxamide (BIP)_2_B.

Belon et al. recently described how a prototype in the symmetrical benzimidazolephenyl series, the *N^1^*,*N^4^*-bis[4-(1*H*-benzimidazol-2-yl)phenyl]benzene-1,4-dicarboxamide (BIP)_2_B, (**69a**, derivative of **69** with Y = phenyl, Figure 18), binds directly the HCV NS3 helicase in the same binding site for RNA in a competitive manner. Furthermore, they reported that **69a** interacts with NS3 encoded non only by various HCV genotypes, but even by Dengue virus (DV), Japanese encephalitis virus (JEV) and, even if less tightly, the related human helicase [72].

Also small peptides specifically inhibit HCV helicases, even in cells bearing HCV replicon. Between them, a peptide expressed in bacteria, composed of 14 amino acids (**p14**, RRGRTGRGRRGIYR), demonstrated to be the best enzyme inhibitor. **P14** has the same amino-acidic sequence as that surrounding the putative HCV helicase arginine finger and inhibits the replication of HCV replicon in cells with an EC_50_ = 83 *μ*M [73], while reduces the DNA unwinding with an IC_50_ of 0.2 *μ*M [74]. 

**Figure 19:**
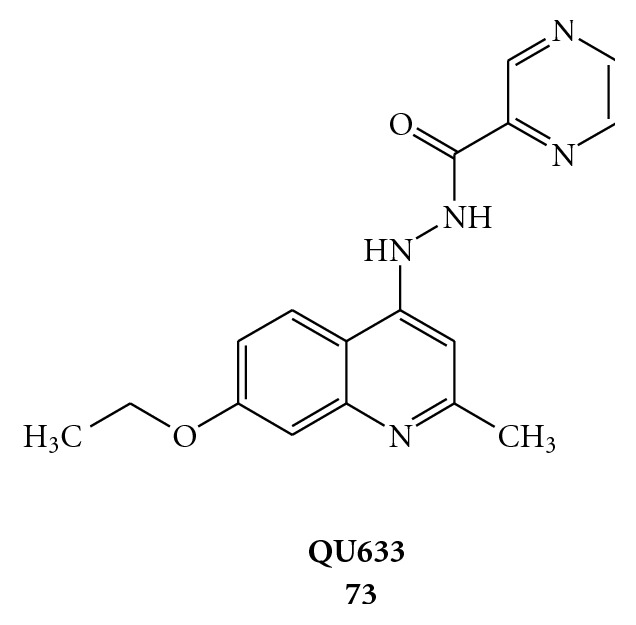
Molecular formula of (N′-(pyrazinecarbonyl)-N′′-(7-ethoxy-2-methylquinolin-4-yl)hydrazine) (QU633).

A new selective inhibitor of the HCV helicase, QU663 (compound **73** of Figure 19), discovered by Maga and coworkers, showed a potent and selective inhibition with Ki of 0.75 *μ*M  [75]. The study of the inhibition mechanism has revealed that QU663 is a specific inhibitor of the strand-displacement activity, without affecting the ability of NS3 helicase to hydrolyse ATP. QU663 could function as a competitive inhibitor with respect to nucleic acid substrate by decreasing the affinity of the enzyme for the substrate. Molecular docking studies further support this explanation. Therefore, QU663 inhibits the unwinding activity of NS3 in a competitive manner with respect to the DNA substrate, making it a promising candidate for a novel class of anti-HCV drugs.

**Figure 20:**
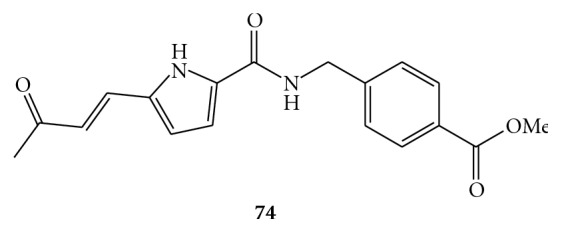
Molecular structure of compound **74.**

Recently, a new rational approach for the design of selective inhibitors of the HCV NS3 helicase brought the discovery of a novel HCV helicase inhibitor that potentially could compete for the nucleic acid binding site, occupying the NS3/RNA binding cler. In consequence of this *de novo* drug design, the predicted (*E*)-methyl 4-((5-(3-oxobut-1-enyl)-1*H*-pyrrole-2-carboxamido)methyl)benzoate (**74**, Figure 20) was synthesized and tested in the HCV replicon system. It inhibits HCV replicons with an EC_50_ of 9 *μ*M, but showing a CC_50_ = 30 *μ*M [76].

### 3.4. Inhibition of the Unwinding through Intercalation of Polynucleotide Chain

DNA and RNA intercalating compounds are potential helicase inhibitors by increasing the energy required for duplex/intercalator complex unwinding [77–79].

**Figure 21:**
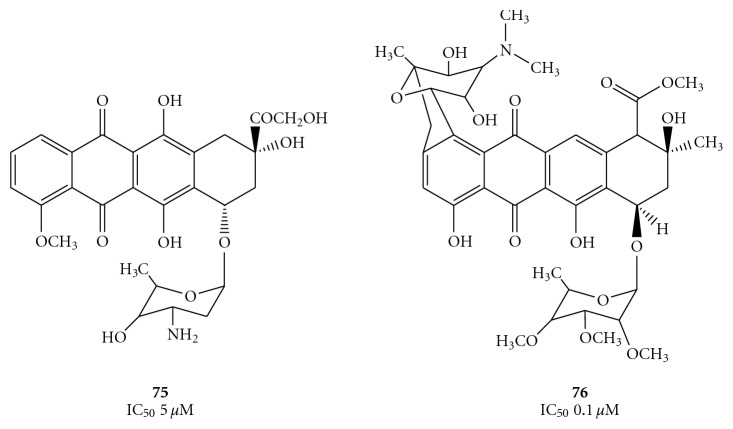
Structures of two DNA/RNA intercalators doxorubicin (**75**) and nogalamycin (**76**) that have displayed inhibition of the unwinding reaction catalyzed by HCV helicase.

In particular, two anthracycline derivatives, doxorubicin and nogalamycin (compounds **75** and **76**, Figure 21), have been shown to be effective inhibitors of the unwinding reaction [77]. The limits in their application for the treatment of chronic viral infections is their high cytotoxicity and weak penetration into the cell. Thus, if intercalative modulation of the DNA or RNA substrates is to be considered as a possible antiviral therapy, less toxic and more selective derivatives must be identified.

As previously seen, the antibiotic nogalamycin (**76**), that interacts with allosteric binding site, has been recently used to obtain a structure-based pharmacophore model for JEV NS3 helicase/NTPase [55]. 

**Figure 22:**
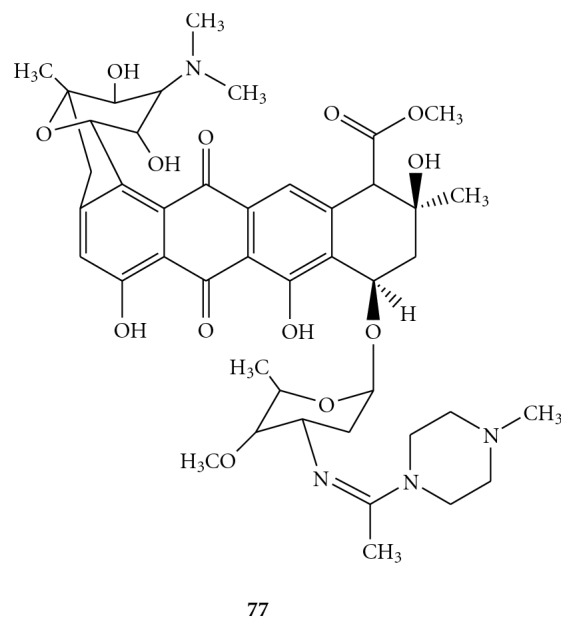
Structural formula of amidinoanthracycline derivative **77.**

In the aim to find less toxic compounds, a large group of amidinoanthracyclines, with decreased acute toxicity and cardiotoxicity compared to the parent antibiotics, were screened against HCV helicase. From this studies emerged one of the most potent and selective inhibitors of helicase activity described in the literature. The derivative **77**, showed in Figure 22, acts not only *via* intercalation into the double-stranded DNA substrate, but also impeding formation of the active helicase complex *via* competition with the enzyme for access to the substrate. Tested in the subgenomic HCV replicon system, **77 **it showed an EC_50_ of 0.13 *μ*M and a CC_50_ = 4.3 *μ*M [80].

**Figure 23:**
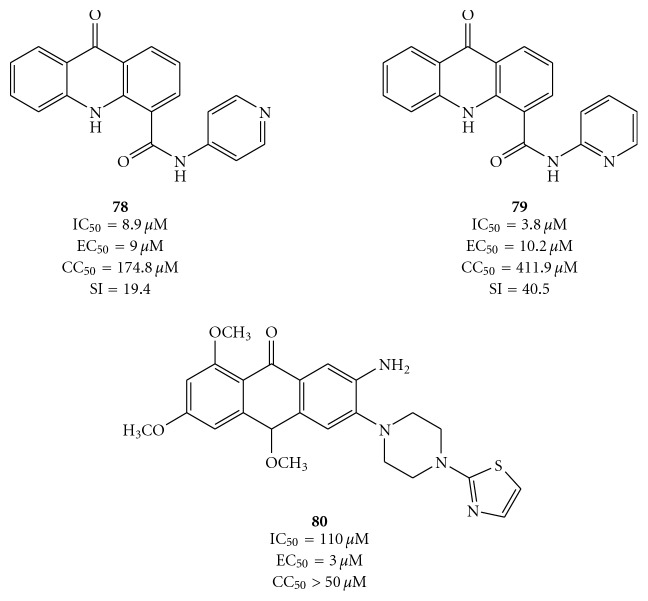
Structures and activity of acridone-4-carboxylic acid derivatives **78** and **79** and of 7-amino-1,3,10-trimethoxy-6-(4-(thiazol-2-yl)piperazin-1-yl)acridin-9(10*H*)-one **80.**

An other class of compounds that probably acts *via* intercalation into double-stranded nucleic acids with strong specificity for RNA are the acridone derivatives, but a direct interaction with the viral NS3 helicase cannot be excluded. A large group of acridones were tested from Stankiewicz-Drogon et al. using the direct fluorometric helicase activity assay to determine their inhibitory properties towards the NS3 helicase of HCV. From a preliminary study, *N*-(pyridin-4-yl)-amide (**78**) and *N*-(pyridin-2-yl)-amide (**79**) of acridone-4-carboxylic acid emerged to be efficient RNA replication inhibitors with a good specificity in subgenomic replicon system and low cytotoxicity (Figure 23) [81]. Even the thiazolpiperazinyl acridone derivative **80** demonstrated to act as a potent agent against HCV replicons (EC_50_ = 3 *μ*M) and as a selective inhibitor of the HCV NS3 helicase, albeit with low potency (IC_50_ = 110 *μ*M) [82]. Comparing acridone derivatives **78** and **79** with **80**, we can see that the amide bonding formed after the derivatization of acridone-4-carboxylic acid with amines seemed to increase affinity and selectivity for the NS3 enzyme [81].

**Figure 24:**
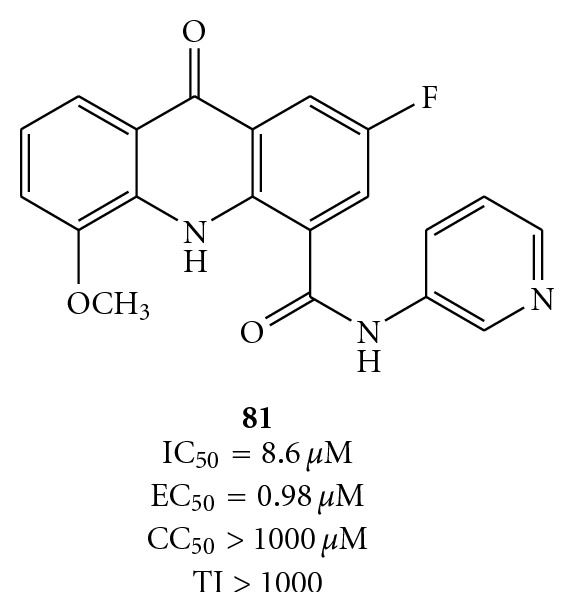
Structures of 2-fluoro-5-methoxy-9-oxo-N-(pyridin-3-yl)- -9, 10-dihydroacridine-4-carboxamide (**81**).

Finally, with the intent to improve the antiviral activity of acridones, Stankiewicz-Drogon et al. prepared a new class of compounds, namely, 5-methoxyacridone-4-carboxylic acids (MACA). From this group, compound **81** (Figure 24) came out not only as an efficient inhibitor of the NS3 helicase in the *in vitro* assay but also as a potent inhibitor of HCV replication endowed with low cytotoxicity for human hepatoma cells [83]. 

## 4. Coronaviridae

**Figure 25:**
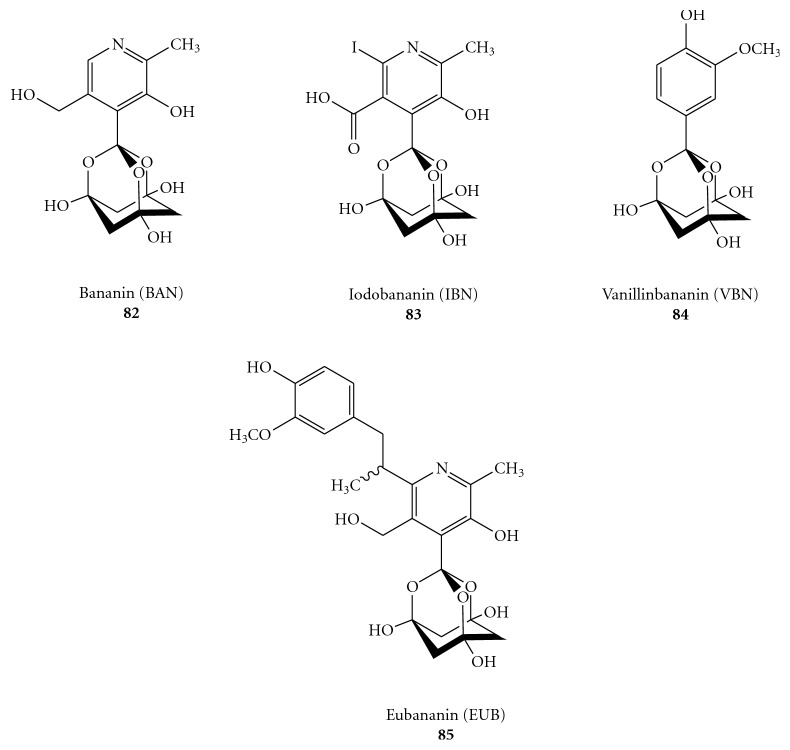
Molecular formula of Bananin (BAN) (**82**), Iodobananin (IBN) (**83**), Vanillinbananin (VBN) (**84**), Eubananin (EUB) (**85**).

An enveloped single-stranded positive-sense RNA (ssRNA^+^) virus, SARS *coronavirus* (SARS-CoV), has been recently identified as the etiological agent of severe acute respiratory syndrome (SARS) in humans [84–88]. About ten thousand cases of SARS worldwide, including 800 deaths, were reported in 2003 (WHO data). Although this initial global outbreak, SARS appears to has been successfully contained, but it remains a serious concern because no vaccine or effective drug treatment is actually available. Recently, Tanner and coworkers have found that Bananin and three of their derivatives, Figure 25, function as non-competitive SARS-CoV helicase inhibitors (with IC_50_ values in the micromolar range) at a site different from the ATP and nucleic acid binding site, causing inhibition probably through an allosteric mechanism [89]. In foetal rhesus kidney-4cells infected with SARS-Cov, Bananin inhibited the viral replication (IC_50_ **=** 10 *μ*M) with low host cellular toxicity (CC_50_ **=** 390 *μ*M) [89].

Finally, in the last years various molecules have been detected showing an interesting and promising anti-Coronaviridae activity. Unfortunately, for many of them, was not identified a clear molecular target or mechanism of action. The fact remains that the eventual target could be the NS3 helicase. With this in mind, we report briefly the new classes of compounds that have emerged in recent published works. Among them glycopeptide antibiotics [90], which seem to interfere with the Coronavirus entry process but do not exclude an unknown cellular target; pyridine N-oxide derivatives [91]; plant lectins [92], which most probably interfere with the glycans on the spike protein during virus entry and virus release; phenanthroindolizines and phenanthroquinolizidines [93]; tetrahydroquinoline oxocarbazate derivatives as inhibitor of human cathepsin L and as entry blockers [94].

## 5. Picornaviridae

Picornaviridae family includes 9 genera, 3 of which are human pathogens: Enterovirus (containing poliovirus, enterovirus, coxsackievirus, echovirus), Rhinovirus (approximately 105 serotypes), and Hepatovirus (*Hepatitis A virus*). At present no specific antiviral therapy is available for the treatment of Picornaviridae infections. The viruses belonging to this family, all having a single-stranded positive-sense RNA (ssRNA^+^) genome, cause a dramatic variety of illnesses, including meningitis, colds, heart infection, conjunctivitis, and hepatitis.

**Figure 26:**
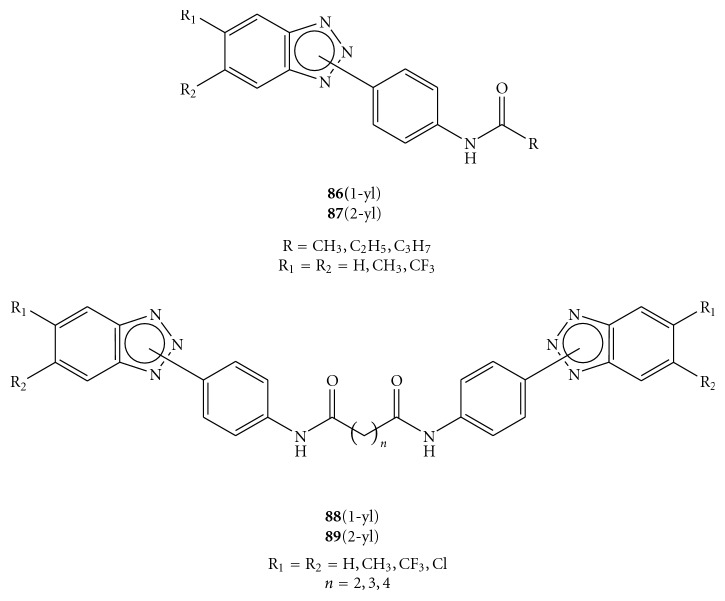
Molecular formula of novel *N*-[4-(1*H*(2*H*)-benzotriazol-1(2)-yl)phenyl]alkylcarboxamides (**86**–**87**) and *N,N′*-bis-[4-4-(1*H*(2*H*)-benzotriazol-1(2)-yl)phenyl]alkylcarboxamides (**88**–**89**).

Recently Carta and coworkers reported the synthesis and antiviral screening of a series of *N*-[4-(1*H*(2*H*)-benzotriazol-1(2)-yl)phenyl]alkylcarboxamides  (**86**(1-yl), **87**(2-yl) [95] and *N,N′*-bis-[4-(1*H*(2*H*)-benzotriazol-1(2)-yl)phenyl]alkyldicarboxamides (**88**(1-yl),** 89**(2-yl)) [78] (see Figure 26).

**Figure 27:**
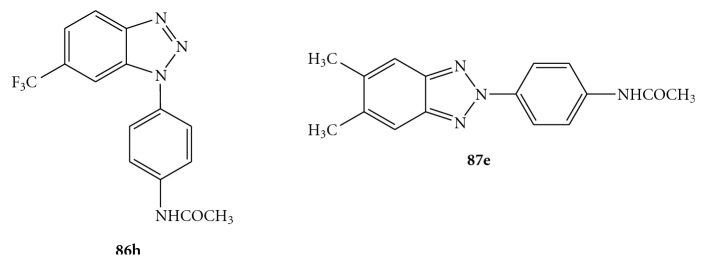
Molecular formula of compounds **86h** and **87e**.

**Figure 28:**
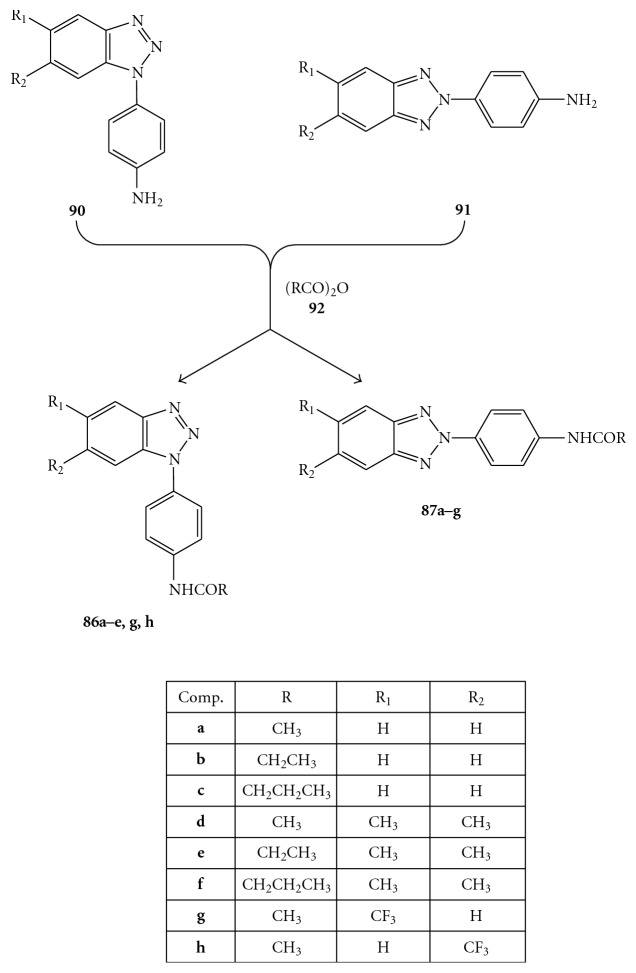
Synthesis of *N*-[4-1*H*(2*H*)-benzotriaol-1(2)-yl)phenyl]alkylcarboxamides (**86** and **87**).

**Figure 29:**
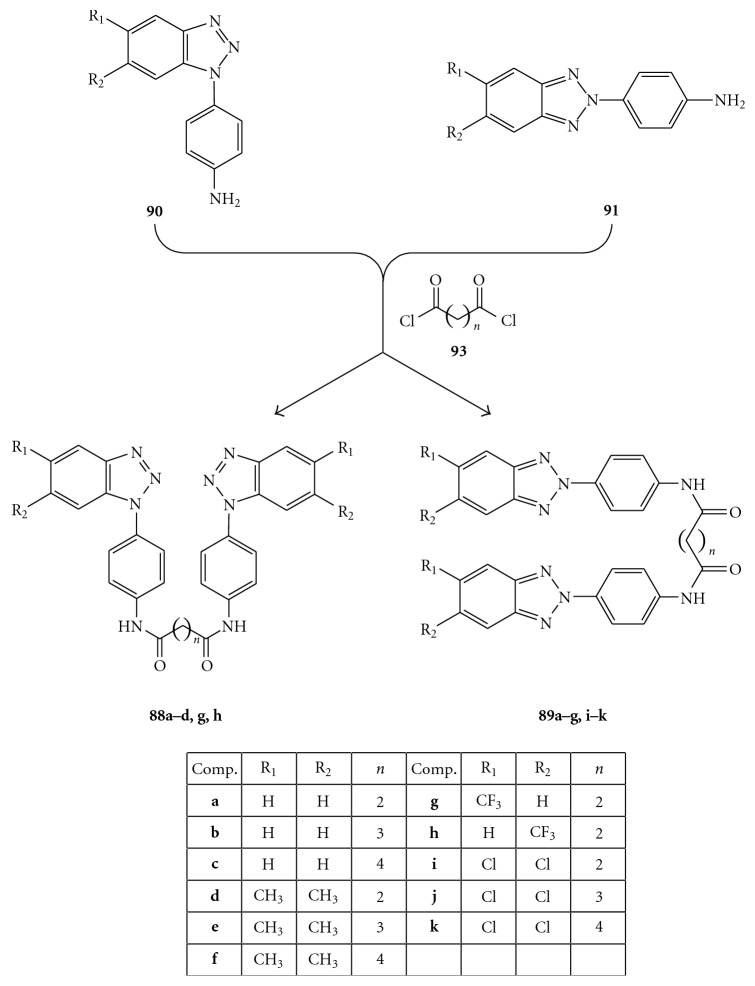
Synthesis of *N,N′*-bis[4-1*H*(2*H*)-benzotriaol-1(2)- yl)phenyl]alkylcarboxamides **88a**–**d**, **g**, **h** and **89a**–**g, i**–**k.**

Compounds were evaluated *in vitro* for cytotoxicity and antiviral activity against a wide spectrum of ssRNA^+^ viruses, like Bovine Viral Diarrhea Virus (BVDV), Yellow Fever Virus (YFV), Coxsakie Virus B (CVB-2), Polio Virus (Sb-1), and Human Immunodeficiency Virus (HIV-1). Only CVB-2 and Sb-1 were inhibited by *N*-[4-(1*H*(2*H*)-benzotriazol-1(2)-yl)phenyl]alkylcarboxamide derivatives. In particular, two of them emerged for their selectivity: **87e**, which was the most active against CVB-2 (EC_50 _= 10 *μ*M and CC_50 _> 100 *μ*M) and **86h,** which was the most active against Sb-1 (EC_50_ = 30 *μ*M and CC_50_ = 90 *μ*M), Figure 27 [95]. *N*-[4-(1*H*(2*H*)-benzotriazol-1(2)-yl)phenyl]alkylcarboxamides (**86a**–**e**,**g**,**h **and** 87a**–**g**) were prepared by condensation of the amino derivatives **90**, **91** with the appropriate anhydrides **92** under stirring at 100°C for 2 h, as shown in Figure 28. The  *N*,*N′*-bis[4-(1*H*(2*H*)-benzotriazol-1(2)-yl)phenyl]alkyldicarboxamides  (**88a**–**d**,  **g**,  **h**  and  **88a**–**d89a**–**g**,  **i**–**k**)  were in turn prepared, as reported in Figure 29, by condensation of the amines 1(2)-(4-aminophenyl)benzotriazoles (**90, 91**) with the suitable diacyl dichlorides (**93**). 

Among  *N*,*N′*-bis-[4-(1*H*(2*H*)-benzotriazol-1(2)-yl)phenyl]alkyldicarboxamides,  the  bis-5,6-dimethyl-derivatives  (**89d**–**f**)  exhibited  good  activity  against Enteroviruses (EC_50_ were 7–11 *μ*M against CVB-2 and 19–52 *μ*M against Sb-1) and the bis-5,6-dichloro-benzotriazol-2-yl derivatives (**80i**–**k**) showed very selective activity against CVB-2 (EC_50_ = 4–11 *μ*M) resulting to be completely inactive against all the other viruses screened [96].

**Figure 30:**
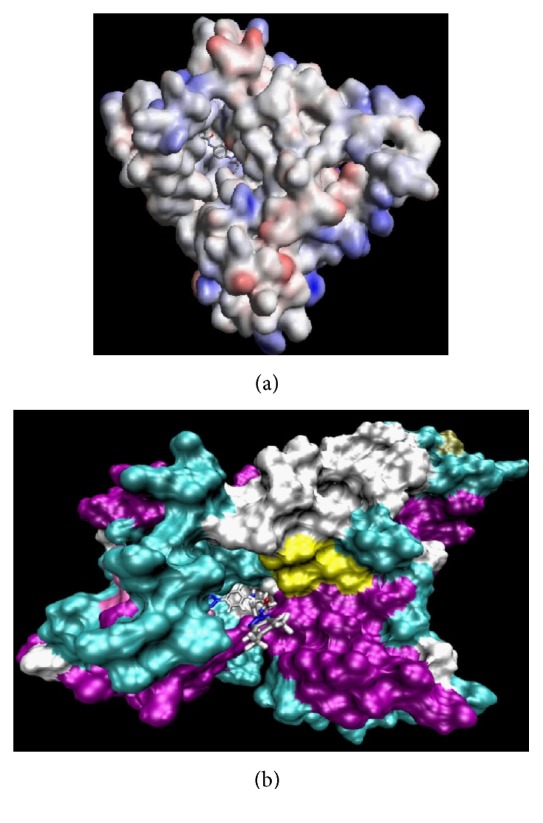
Binding of compounds **86h** (a) and **89f** (b) to the putative binding site on the surface of Polio (Sb-1) helicase.

*N*-[4-(1*H*(2*H*)-benzotriazol-1(2)-yl)phenyl]alkylcarboxamides (**86 **and** 87**) were evaluated *in silico* against the 3D model of the Sb-1 helicase, as exemplified by compounds **86h** (a) and **89f** (b) in Figure 30. The portion of the enzyme containing the binding site interacting with the inhibitors consists of two loops, part of two *β*-sheets, and part of three helices. 

It is important to notice that, with respect to the *N*-[4-(1*H*(2*H*)-benzotriazol-1(2)-yl)phenyl]alkylcarboxamide series,  all  the  *N*,*N′*-bis[4-(1*H*(2*H*)-benzotriazol-1(2)-yl)phenyl]alkyldicarboxamide derivatives bind helicase Sb-1 in a different manner, as expected, due to their different shapes and dimensions. This is well quantified by the value of the solvent accessible volume of this new class of inhibitors, which on average has almost doubled compared to that of the previous molecular series (e.g., 1672 Å^3^ versus 891 Å^3^, resp.). Accordingly, it is impossible for the protein pocket to host *N,N′*-bis[4-(1*H*(2*H*)-benzotriazol-1(2)-yl) phenyl]alkyldicarboxamides with the same binding mode, and the results form the docking study reveal that only one of the two identical inhibitors moieties can be positioned well within the binding pocket. However, in correspondence of the most favored binding mode for the most active compounds, the formation of a new, small network of H-bonds between **89f **and enzyme was observed. In particular, the analysis of the trajectories of the MD simulations for the **89f**/helicase complex as an example indicates that there is a constant presence of an H-bond which involves the carbonyl oxygen atom of the Asn179 side chain and the triazole N(1) atom of the drug, characterized by an average dynamic length (ADL) of 3.0 Å. At the same time, it is possible to verify the formation of other two H-bond interactions, the former between the C=O backbone group of Ser221 and the NH group of the amidic moiety of **89f **(ADL = 1.6 Å), and the latter between the carbonyl oxygen atom of the C=O group of the same amidic moiety of **89f **and the side chain hydroxyl group of Ser221 (ADL = 2.6 Å).

Compounds **89i**–**k** also exhibited selectivity against Coxsackie B2CVB-2. Unfortunately, homology standard techniques were not able to produce a reliable 3D model for the CVB-2 virus helicase, due to very low sequence identities found during alignment processes.

In the absence of a 3D model for the CVB-2 helicase, the activity of **89i**–**k** can be explained adopting a 2D alignment analysis.

The putative binding site proposed by Carta and coworkers for Sb-1 is composed by 30 residues and, according to their 2D alignment, the binding site for CVB-2 differs for 7 residues only. Among these, Ser296 in Sb-1 is mutated to Arg237 in CBV-2. Following their analysis, and a preliminary visual inspection based on the swapping of Ser to Arg in the Polio helicase, they concluded that this is the most important residue in the case of compounds **89i**–**k**, featuring chlorine atoms as substituents. In fact, the positively charged side chain of Arg237 is placed at an average distance of 3.5 Å from the Cl atoms, thus sensibly resulting is strong electrostatic interactions between the inhibitor and the protein. These speculations, which may account in part for the selectivity of these compounds with respect to CVB-2, clearly await further confirmation from the simulations performed on the corresponding protein 3D models.

## Figures and Tables

**Table 1:** Viral helicases of same ssRNA^+^ Viruses (belonging to Flaviviridae, Coronaviridae, and Picornaviridae families) [9, 10, 33].

Family	Species	Helicase family	Helicase name	*In vitro *activity
Flaviviridae	*Yellow fever virus*	**SF2**	NS3	RNA stimulated NTPase
	*West Nile virus*	**SF2**	NS3	RTPase 3′–5′ helicase
	*Dengue fever virus*	**SF2**	NS3	3′–5′RNA helicase RTPase
	*Japanese encephalitis virus*	**SF2**	NS3	3′–5′RNA helicase
	*Bovine viral diarrhea virus*	**SF2**	NS3	3′–5′RNA helicase
	*Hepatitis C virus*	**SF2**	NS3	3′–5′RNA /DNA helicase
	*Hepatitis G virus*	**SF2**	NS3	3′–5′RNA /DNA helicase
	*Hepatitis A virus*	**SF3**	2C	NTPase
Coronaviridae	*Human coronavirus 229E*	**SF1**	Nsp 13	3′–5′RNA/DNA helicase RTPase
	*SARS Coronavirus*	**SF1**	Nsp 13	3′–5′RNA/DNA helicase RTPase
Picornaviridae	*Poliovirus*	**SF3**	2C	NTPase
	*Rhinovirus*	**SF3**	2C	NTPase
